# Optical and polarization properties of nonpolar InGaN-based light-emitting diodes grown on micro-rod templates

**DOI:** 10.1038/s41598-019-46343-0

**Published:** 2019-07-05

**Authors:** J. Bai, L. Jiu, N. Poyiatzis, P. Fletcher, Y. Gong, T. Wang

**Affiliations:** Department of Electronic and Electrical Engineering, University of Sheffield, Mappin Street, Sheffield, S1 3JD United Kingdom

**Keywords:** Lasers, LEDs and light sources, Materials for devices

## Abstract

We have demonstrated non-polar *a*-plane InGaN multiple-quantum-well (MQW) light-emitting diodes (LEDs) on sapphire, achieved by overgrowing on a micro-rod template with substantially improved crystal quality. Photoluminescence measurements show one main emission peak at 418 nm along with another weak peak at 448 nm. Wavelength mapping measurements carried out by using a high spatial-resolution confocal PL system indicate that the two emissions origin from different areas associated with the underlying micro-rod patterns. Electroluminescence measurements exhibit a negligible blue-shift of 1.6 nm in the peak wavelength of the main emission when the driving current increases from 10 to 100 mA, indicating that the quantum confined Stark effect is effectively suppressed in in the nonpolar LED. A polarization ratio of 0.49 is obtained for the low-energy emission (~448 nm), while the main emission (~418 nm) shows a polarization ratio of 0.34. Furthermore, the polarization ratios are independent of injection current, while the energy separation between m-polarized and c-polarized lights increases with the injection current for both emissions.

## Introduction

III-nitride semiconductors grown along nonpolar orientations have attracted extensive interest in recent years, due to a few unique properties in comparison with their c-plane polar counterparts, especially for InGaN-based emitters. One major advantage of nonpolar InGaN emitters is absence of polarization-induced electric field along the growth direction, which exist in *c*-plane InGaN emitters. The large internal field results in the well-known quantum-confined Stark effect (QCSE) and reduced overlapping of electron and hole wavefunctions, thus leading to reduced quantum efficiency^1–3^. A few approaches are employed to improve the overlap by engineering the quantum well active layers, including introducing indium in the barriers^4^ or step-function like indium content in the well^5^. When nonradiative recombination is suppressed properly through the growth on bulk GaN substartes and special design, high quantum efficiencies (~80%) can be achieved for *c*-plane InGaN light emitting diodes (LEDs)^6^. Nevertheless, one of the most promising solutions to fundamentally solve the issue is growing nonpolar GaN. More importantly, nonpolar InGaN emitters exhibit another major advantage which current c-plane ones lack, their polarized light source, which results from the valence band splitting generated by anisotropic biaxial stress^7,8^. This will play an important role in manufacturing backlighting in terms of improving power consumption and compactness^9^, as current approach is to insert a polarizer and thus up to 30% optical power is wasted.

So far, high performance nonpolar InGaN emitters are basically grown on expensive GaN substrates normally with very small sizes of 10 × 10 mm^2 ^^10,11^. It is therefore highly expected to achieve high quality nonpolar GaN on industry-matched substrates, such as sapphire or silicon. However, the nonpolar GaN directly grown on either sapphire or silicon have a high density of defects, with a dislocation density of above 10^10^/cm^2^ and a stacking fault density of above 10^6^/cm^12,13^. Patterned sapphire approach is employed to the growth of non-polar GaN, leading to improvement in crystal quality^14^. The main difficulty remains in etching a specific grooved sapphire where facets with an accurate inclination angle are strictly required for the growth of desirable nonpolar GaN, simultaneously avoiding a corrugated surface morphology with micro-facets^15^. Moreover, epitaxial lateral overgrowth approaches have been also extended to the nonpolar GaN growth on planar sapphire^16–18^, nomally employing stripe-patterned templates. However, it is usually difficult to achieve an atomically flat surface on such a stripe-patterned template due to the intrinsically anisotropic in-plane growth rate^19^. Using an array of hexagonal SiO_2_ patterns, high quality *a*-plane GaN have been achieved on sapphire substrates. Up to date, output powers of a few mini Watts at 20 mA have been reported for nonpolar InGaN LEDs grown on sapphire, though they are still much lower compared to *c*-plane polar LEDs^20,21^.

**Figure 1:**
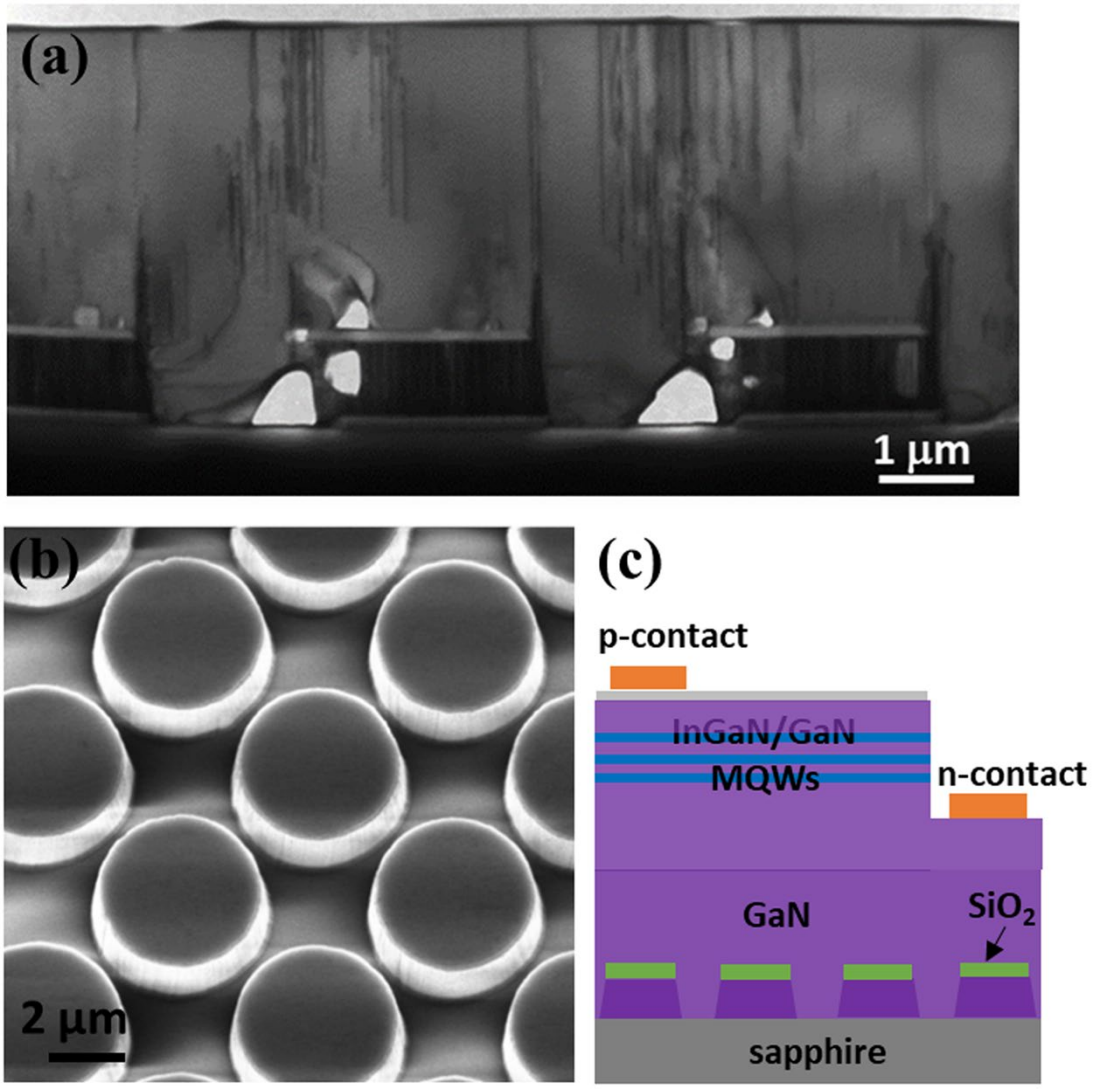
(**a**) Cross-section TEM image of the overgrown *a*-plane GaN on a micro-rod template, taken around [1–100] zone-axis with **g**=<11–22>. (**b**) SEM image of a micro-rod GaN template. (**c**) Schematic of the *a*-plane InGaN/GaN MQW LED.

Our group has achieved high quality nonpolar (11–20) GaN films by using an overgrowth approach on regularly arrayed (11–20) GaN micro-rods on *r-plane* sapphire^19^. The X-ray rocking curves demonstrate the line widths are 270 arcsec along the *c*-direction and 380 arcsec along the *m*- direction, which is the best report so far. The dislocation density in the overgrown GaN is dramatically decreased by two orders in comparison with the as-grown (11–20) GaN, as shown by transmission electron microscopy observation (Fig. 1(a)). In this work, we demonstrate nonpolar InGaN/GaN multiple quantum well (MQW) LEDs grown on such high quality nonpolar GaN templates. Optical and polarization properties are investigated by photoluminescence (PL), Confocal PL, and polarized electroluminescence (EL) measurements.

## Methodology

A standard nonpolar GaN layer is first grown on *r*-plane sapphire by a standard metal organic chemical vapour deposition using our high temperature AlN buffer technology^22^. Next, the nonpolar GaN template is fabricated into regularly arrayed micro-rods using photolithography and dry-etching processes (Fig. 1(b)). In the photomask, both the diameter and spacing of micro-rods are designed to be 2.5 μm. To make sure an easier coalescence for the overgrowth, the micro-rods are formed deliberately to be larger than 2.5 μm during the processing by adjusting the exposure time. The heights of micro-rods is about 1 μm. The micro-rod template then undergoes ultra-violet assisted photo-enhanced chemical etching processes (details refer to ref.^19^), forming a mushroom configuration. The specially designed patterning can compensate for the intrinsically anisotropic in-plane growth rate of nonpolar GaN^23^. Consequently, the overgrowth on the template can achieve not only substantially improved crystal quality, but also an atomically flat surface as result of a quick coalescence.

Following the growth of high quality overgrown nonpolar GaN with a layer thickness of 3 μm, a simple LED structure is grown. The structure includes a 1 μm *n*-type GaN layer, three periods of InGaN/GaN MQWs with 3 nm thick well and 10 nm thick barrier, and 150 nm *p*-type GaN layer. The indium composition in the wells is about 12%. As shown in Fig. 1(c), lateral LEDs with a mesa area of 0.1 mm^2^ were fabricated by photolithography and dry etching. Transparent *P*-type contact was formed by depositing 100 nm ITO which was annealed in air. Ti/Al/Ti/Au alloys were deposited as *n*-type contact. Finally, Ti/Au was deposited as *p*-type and n-type electrodes.

## Results and Discussion

**Figure 2:**
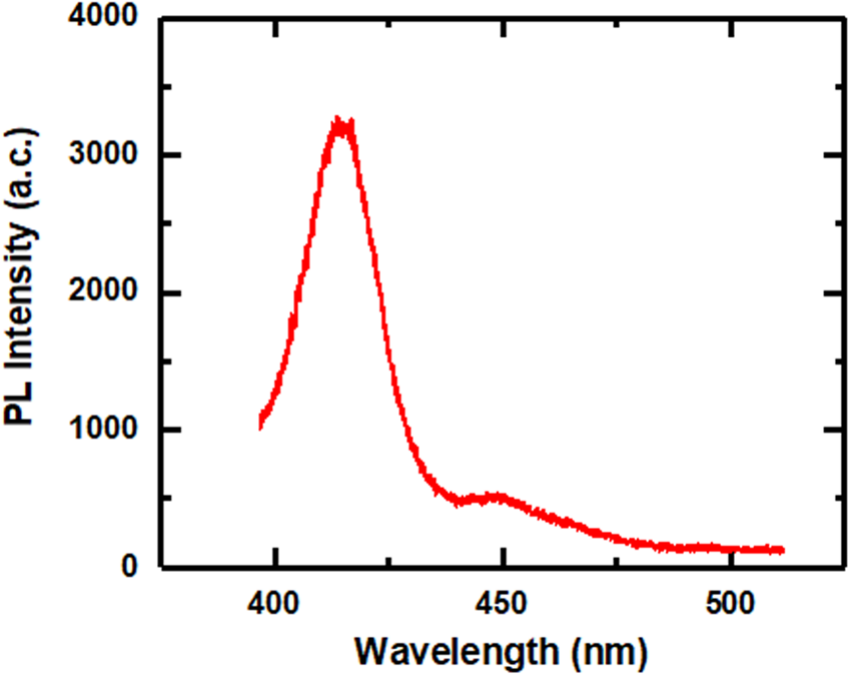
PL Emission spectra of the nonpolar InGaN/GaN MQW LED at room temperature.

**Figure 3:**
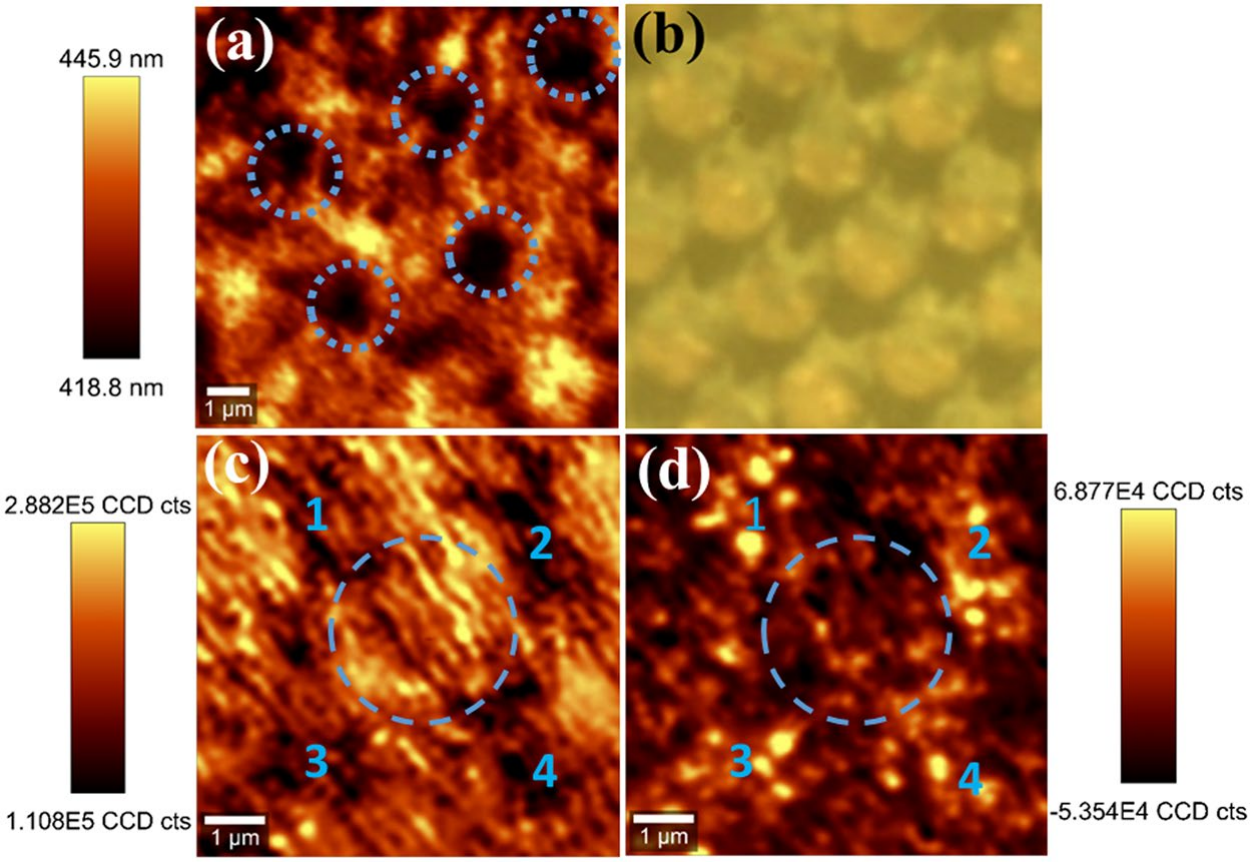
A wavelength map of confocal PL (**a**), and a microscope photo **(b**) for the nonpolar LED. The intensity maps of confocal PL with 418 nm (**c**) and 448 nm (**d**) emission for the nonpolar LED.

Standard photoluminescence (PL) measurement is performed at room temperature using a 375 nm diode laser to excite the nonpolar LED sample, with the luminescence dispersed by a 0.55 m monochromator and detected by a charge-coupled device (CCD). The laser power is 29 mW with a spot diameter of 0.1 mm, leading to an excitation power density of 369 W/cm^2^. The spectrum of the nonpolar LED sample is shown in Fig. 2(a). It shows that the emission is dominated by a peak at 418 nm, with another weak emission appearing on the longer wavelength side at 448 nm. In order to investigate the origin of the double emission peaks from the InGaN/GaN MQWs, confocal PL measurements are carried out using a 375 nm laser source with a WiTec confocal microscopy and an optical microscope system, where the laser beam has been focused into a diameter of 200 nm with an excitation power of 0.3 mW. The emission is collected into a Horiba CCD, through a 300 nm grating. Figure 3(a) shows a typical wavelength mapping within an area of 10 μm × 10 μm. The scale bar shows the PL wavelength ranging from 418.8 nm to 445.9 nm. The color pattern in Fig. 3(a) indicates an emission wavelength distribution. Please note that such a color pattern formed is similar to the micro-rod pattern photo taken in Fig. 3(b). Comparing with the micro-rod pattern in Fig. 3(b), it is found the dark regions in the map marked by dashed circles correspond to the areas just above micro-rods, while the bright regions correspond to the areas between the micro-rod gaps. Furthermore, the 7 μm × 7 μm intensity maps, which focus around one micro-rod, are taken using a filter of 418 nm and of 448 nm. In the map corresponding to the 418 nm emission (Fig. 3(c)), the intensity is generally high across the map, except that a few positions around the micro-rod are dark, marked by 1, 2, 3 and 4. In contrast, these positions show the largest intensities in the map corresponding to the 448 nm emission (Fig. 3(d)). It indicates that the indium distribution in the InGaN/GaN MQWs is related to the micro-rod patterned template for the GaN overgrowth, though a detailed investigation on the mechanism of indium composition variation is in progress. It is worth mentioning that the dual emission of our nonpolar LEDs is an advantage in achieving nonpolar white LEDs as a result of the multiple colour emissions.

**Figure 4:**
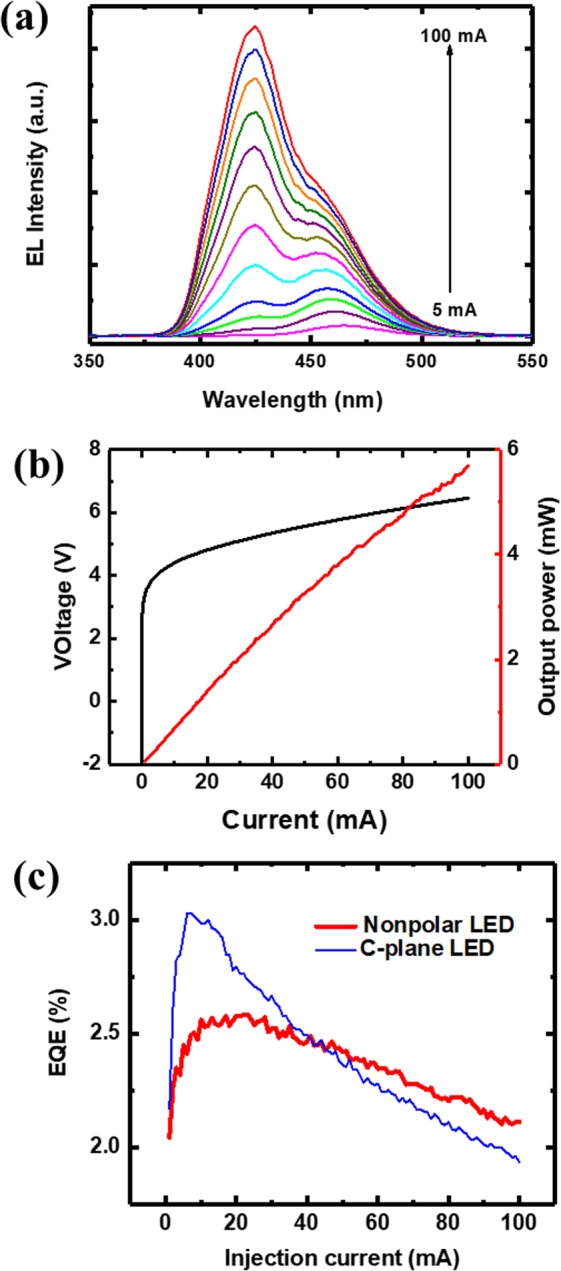
(**a**) EL spectra measured at different injection currents, and **(b**) L-I-V characteristic of the non-polar LED. (**c**) EQE of the LED as a function of current from 5 mA to 100 mA. A c-plane LED (dashed line) is measured as a reference.

Electroluminescence (EL) measurements are performed on bare-chip devices at room temperature in a cw mode with a Keithley 2400 source meter at a microscope station. Figure 4(a) shows the EL spectra under different injection currents. At low currents, only the emission with the longer wavelength is observed. The main emission with the shorter wavelength appears at higher currents, and the intensity increases with the increase of the injection current. At 100 mA current, the emission with the shorter wavelength becomes dominant over the spectrum. When the driving current is low, recombination has more chances of taking place at the QW energy band with a lower energy. At higher driving currents when the lower energy band is filled, excitons start to diffuse into the QW energy band with a higher energy. It is important to note, the wavelength of the main emission shifts only about 1.6 nm with increasing the current from 10 mA to 100 mA. It indicates suppression of piezoelectric field-induced QCSE in the nonpolar InGaN LED, and that the piezoelectric field is nearly eliminated in the nonpolar LED. Moreover, the output powers are measured on the bonded bare-chip LEDs in a cw mode, using a LCS-100 characterization system equipped with an integrating sphere and a CCD APRAR spectrometer. The output power-current-voltage (L-I-V) characteristic is displayed in Fig. 4(b), showing good electrical property of the non-polar LED. The light power increases lineally with increasing the current without any saturation tendency with an output power of 1.3 mW at 20 mA. Furthermore, the external quantum efficiency (EQE) has been calculated as a function of current as well, as shown in Fig. 4(c). In order to compare the efficiency performances of the nonpolar LED to *c*-plane LEDs, the EQE of a fabricated blue *c*-plane LED grown on sapphire is also displayed by one dashed line in Fig. 4(c) as a reference. It shows that the EQE of the *c*-plane LED has a maximum value of 3.9% at a very low current (~6 mA), and then quickly decreases till 1.9% at 100 mA. In contrast, after reaching a maximum of 2.6% at 23 mA, the EQE of the nonpolar LED shows a very slow decrease with further increasing the current. In detail, the EQEs at 100 mA drop down to 82% and 63% of their maxima for the nonpolar LED and the c-plane LED, respectively. Note that both the c-plane LED and the nonpolar LED are grown on sapphire with an identical simple LED structure. Although the EQEs of the *c*-plane LED at low currents are higher in comparison with the nonpolar LED, the nonpolar LED demonstrates a much more stable efficiency with the change of the current and maintains higher EQE values at high currents. It indicates that the efficiency-droop of InGaN LEDs can be improved through growth and fabrication of nonpolar ones.

**Figure 5:**
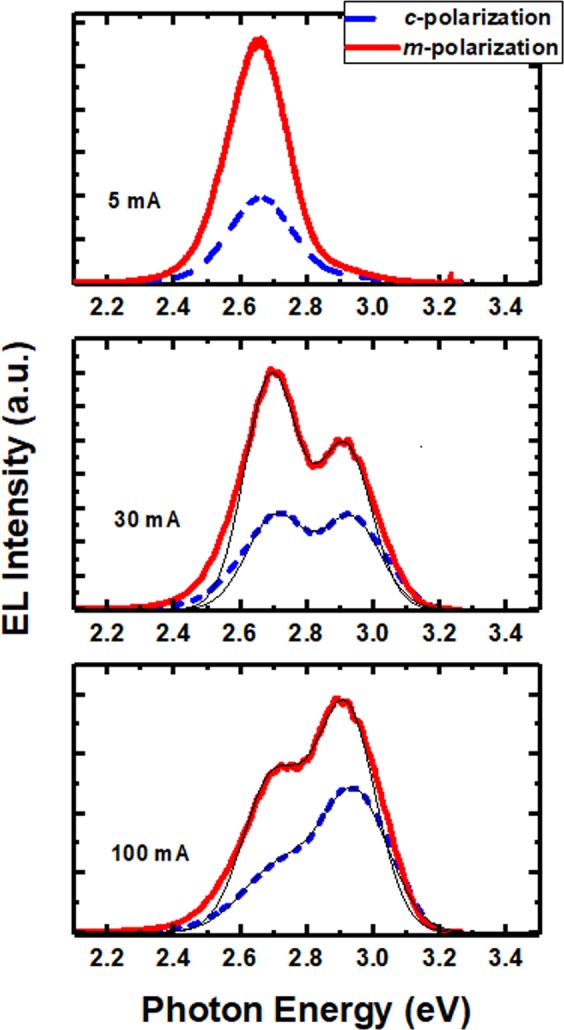
EL spectra with *c*-polarization and *m*-polarization at 5 mA, 30 mA and 100 mA. The thin black lines show the Gaussian curve fittings to determine the emission energies.

**Figure 6:**
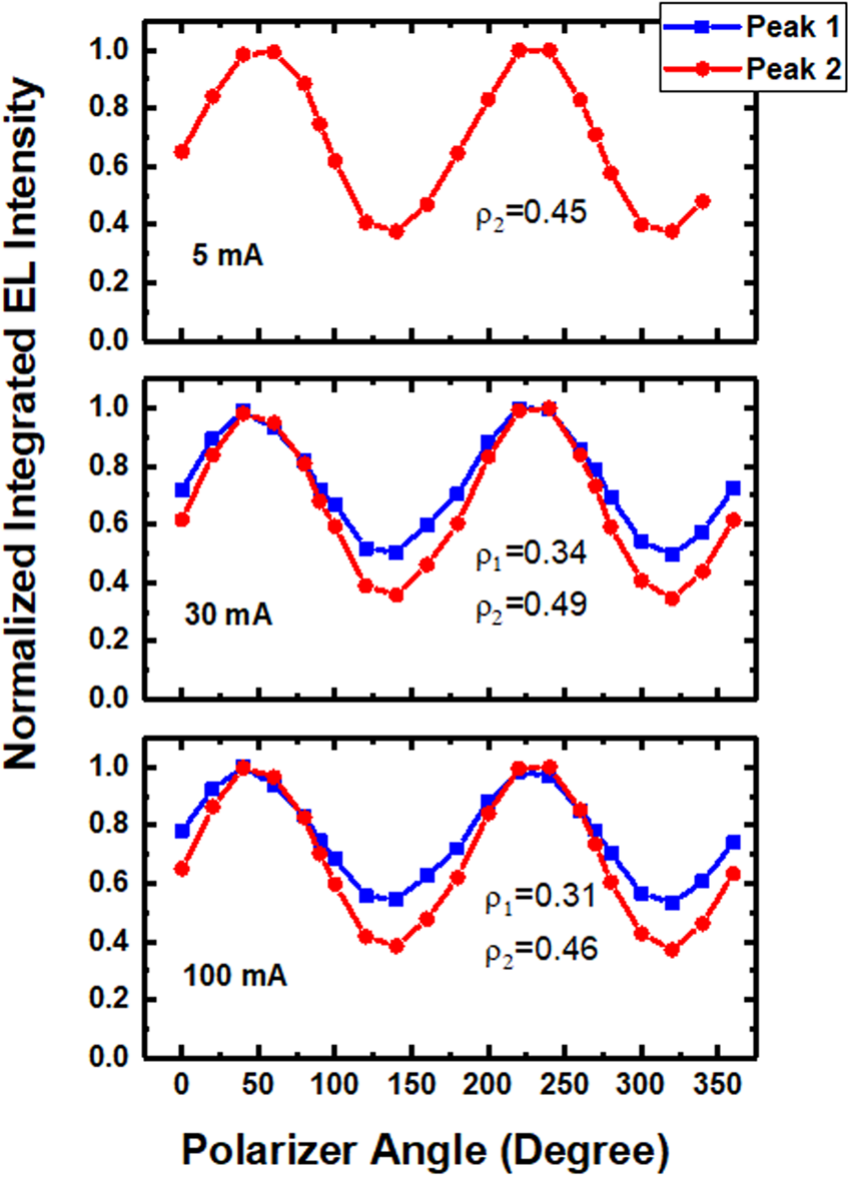
Polarization ratios for the 418 nm (peak 1) and 448 nm (peak 2) emissions at 5 mA, 30 mA and 100 mA.

As we know, due to anisotropic strain, original valence band of a nonpolar InGaN/GaN QW system is broken into the |X>, |Y>, and |Z> sub-bands, among which the |X> state and the |Y> state have the smallest and largest electron energies, respectively^7,24^. The transition from conduction band to the |Y> state results in the light with polarization parallel with *m*-direction (perpendicular to *c*-direction), whereas the transition involving the |Z> state leads to the higher energy light with polarization parallel with *c*-direction. To assess the polarization properties of the non-polar LED, polarized EL spectra are measured from the top surface of the LED by rotating a polarizer positioned between the device and a spectrometer^25^. Before the experiments, the *c*-plane LEDs have been proven to have negligible polarization characteristics, as a result of careful consideration and calibration of intrinsic polarization of the measurement system. Figure 5 shows the spectra along *c*-polarization and *m*-polarization (with polarization parallel with the *c*-direction and *m*-direction, respectively), measured at different injection currents of 5 mA, 30 mA, and 100 mA. Generally, the EL intensities with *m*-polarization are larger than those with *c*-polarization. Importantly, for the spectra at 30 mA with two emission peaks observed clearly, the difference between the intensities of the *m*-polarized light and the *c*-polarized light is larger for the low-energy peak in comparison with the high-energy peak. It suggests the low-energy peak is more polarized than the high-energy peak. It is attributed to the larger anisotropic in-plane strain induced by higher indium composition, resulting in a larger energy separation between the |*Z*> and |*Y*> states and a higher polarization ratio. Figure 6 shows normalized integrated intensities of nonpolar LEDs as a function of polarization angle. Due to the double emissions, the intensities are integrated across different energy ranges for the main emission (peak 1) and the low-energy emission (peak 2), respectively. Polarization ratio *ρ* is defined as (*I*_*m*_ − *I*_*c*_)/(*I*_*m*_ + *I*_*c*_), where *I*_*m*_ and *I*_*c*_ represent the integrated EL intensities parallel to the *m* and *c* directions, respectively^25^. At 30 mA current, *ρ* is 0.34 and 0.49 for peak1 and peak 2, respectively, which confirms that higher indium composition leads to larger polarization degree. The *ρ* values obtained are comparable with other reports for nonpolar blue LEDs^24,26–28^. Actual polarization degrees for the nonpolar LED should be larger than the values obtained, because no special processing, such as using a confocal microscope or black absorber applied to bottom and side surfaces of devices^8^, were performed in our measurements to reduce light scattering which severely affects the polarization. Furthermore, it is found that the polarization ratios for both emissions are nearly independent of injection current. When the light intensity is integrated across the whole energy range, the *ρ* value of the double emission is found to decrease with increasing the current, due to that the intensity ratio of the two emissions with different *ρ* values is current dependent.

The photon-energy difference Δ*E* between the emission spectra associated with the two polarizations can be used to assess the energy difference between the |Z> and |Y> states^29^. As shown in Fig. 5, the energy difference Δ*E* changes with the injection current. At 5 mA, Δ*E* is not apparent (~4 meV) for peak 2 (the low-energy emission), though a noticeable tail appears on the high-energy side of the *c*-polarized emission through normalized EL spectra. For the spectra at 30 mA and 100 mA with two emission peaks, the peak energies are determined by Gaussian curve fittings drawn by thin black lines. The energy difference Δ*E* at 30 mA are about 18 meV and 12 meV for peak 1 and peak 2, respectively. At 100 meV, Δ*E* of peak 1 and peak 2 are 28 meV and 16 meV, respectively. It means that Δ*E* increases with the driving current for both emissions, which is consistent with other reports on *m*-plane InGaN MQW LEDs^8,28^. It was explained by the anti-crossing of |*Z*> and |*Y*> subbands, or crystal momentum conservation. However, according to the calculation^8^, there is no band mixing for *a*-plane InGaN/GaN QWs along the wave vector *k*_*x*_ direction. It is more likely related to that conservation of the crystal momentum is required when the recombination occurs. Because the effective mass of the |*Y*> state is larger than that of the |*Z*> state, electron transitions to the |*Y*> state with large wave vectors *k* are thence restrained in order for obeying the crystal momentum conservation. With increasing the current when the carriers are filling the states with larger *k*, a faster increase in the transition energy to the |*Z*> state occurs compared to the |*Y*> state, hence resulting in larger Δ*E* values at increased currents.

## Conclusions

In summary, nonpolar *a*-plane InGaN MQW LEDs have been achieved on high quality GaN grown on micro-rod templates. A double emission is observed for the nonpolar LED, which is found to be related to the overlying micro-rod template. The effect of piezoelectric field on optical properties is not observed in the power-dependent EL measurements. The efficiency droop is greatly improved compared to *c*-plane InGaN LEDs. The polarization ratios for both emissions are nearly independent of the driving current, with a larger polarization degree for the emission with higher indium composition. The energy separation between *m*-polarized and *c*-polarized lights increases with the current for both emissions.
